# A Novel Dual HDAC6 and Tubulin Inhibitor, MPT0B451, Displays Anti-tumor Ability in Human Cancer Cells *in Vitro* and *in Vivo*

**DOI:** 10.3389/fphar.2018.00205

**Published:** 2018-03-13

**Authors:** Yi-Wen Wu, Kai-Cheng Hsu, Hsueh-Yun Lee, Tsui-Chin Huang, Tony E. Lin, Yi-Ling Chen, Ting-Yi Sung, Jing-Ping Liou, Wendy W. Hwang-Verslues, Shiow-Lin Pan, Wei-Chun HuangFu

**Affiliations:** ^1^The Ph.D. Program for Cancer Biology and Drug Discovery, College of Medical Science and Technology, Taipei Medical University and Academia Sinica, Taipei, Taiwan; ^2^The Ph.D. Program for Cancer Biology and Drug Discovery, College of Medical Science and Technology, Taipei Medical University, Taipei, Taiwan; ^3^School of Pharmacy, College of Pharmacy, Taipei Medical University, Taipei, Taiwan; ^4^Ph.D. Program in Biotechnology Research and Development, College of Pharmacy, Taipei Medical University, Taipei, Taiwan; ^5^Genomics Research Center, Academia Sinica, Taipei, Taiwan

**Keywords:** microtubule, HDAC6, G2/M arrest, apoptosis, prostate cancer, acute myeloid leukemia

## Abstract

The combination cancer therapy is a new strategy to circumvent drug resistance for the treatment of high metastasis and advanced malignancies. Herein, we developed a synthesized compound MPT0B451 that display inhibitory effect against histone deacetylase (HDAC) 6 and tubulin assembly. Our data demonstrated that MPT0B451 significantly inhibited cancer cell growths in HL-60 and PC-3 cells due to inhibition of HDAC activity. MPT0B451 also markedly increased caspase-mediated apoptosis in these cells. The cell cycle analysis showed mitotic arrest induced by MPT0B451 with enhanced expression of G2/M transition proteins. Moreover, molecular docking analysis supported MPT0B451 as a dual HDAC6 and tubulin inhibitor. Finally, MPT0B451 led to tumor growth inhibition (TGI) in HL-60 and PC-3 xenograft models. These findings indicated that MPT0B451 has dual inhibition effects for HDAC6 and tubulin, and also contributed to G2/M arrest followed by apoptotic induction. Together, our results suggested that MPT0B451 may serve as a potent anti-cancer treatment regimen in human prostate cancer and acute myeloid leukemia.

## Introduction

Histone deacetylases (HDACs) are histone-modifying enzymes participating in transcriptional repression through removal of acetyl groups from target histones. In many cancer cells, HDACs are highly expressed (Yoon and Eom, 2016). Since 2006, FDA has approved several HDAC inhibitors, such as vorinostat (SAHA), romidepsin (FK228), belinostat (PXD101) and panobinostat (LBH589), for treating T-cell lymphoma or multiple myeloma.

Histone deacetylases are grouped into four classes, from class I to class IV, based on their sizes, biological activities and sequence homologies to the yeast HDAC proteins. Class I (HDAC1–3 and 8), class IIa (HDAC4, 5, 7 and 9), class IIb (HDAC6 and 10) and class IV (HDAC11) are enzymatically active via a Zn^2+^-dependent mechanism, whereas class III (SIRT1-7) are NAD-dependent HDAC linked to aging (Saunders and Verdin, 2007; Liu et al., 2016; Wang et al., 2017). The structural organization of HDAC6 contains two tandem catalytic domains (CD1 and CD2), a cytoplasmic retention domain and a zinc-finger ubiquitin binding domain (BUZ), and HDAC6 is located mainly in the cytoplasm (Hubbert et al., 2002; Seidel et al., 2015; Simoes-Pires et al., 2017). In addition to its unique cytoplasmic localization, HDAC6 also specifically deacetylates α-tubulin, the most abundant microtubule component, as well as cortactin and Hsp90α rather than histone proteins (Hubbert et al., 2002; Boyault et al., 2007). Through deacetylation of α-tubulin, HDAC6 regulates microtubule dynamic and in turn participates in microtubule-mediated processes including cell division, migration, and angiogenesis (Aldana-Masangkay and Sakamoto, 2011). Similarly, HDAC6 also modulates actin network to regulate cell motility by deacetylating cortactin, which can bind to F-actin, promote F-actin polymerization and enhance cell migration after HDAC6 mediated deacetylation (Saji et al., 2005; Aldana-Masangkay and Sakamoto, 2011). Therefore, overexpression of HDAC6 has been related to tumor cells invasion and metastasis. In addition, HDAC6 regulates proteasome-dependent protein degradation. Hsp90α, the substrate of HDAC6, and its chaperone proteins can stabilize the anti-apoptotic factors and adjust misfolded protein stress to avoid the client proteins from proteasome degradation (Kawaguchi et al., 2003; Li et al., 2013; Nanduri et al., 2015; Seidel et al., 2015). Consequently, high expression of HDAC6 promotes tumor development not only by facilitating cell metastasis but also by preventing misfolded proteins from degradation (Aldana-Masangkay and Sakamoto, 2011).

α-tubulin and β-tubulin are the two components of microtubules which can dimmerize to form a protofilament and confer polarity on microtubule polymerization. Anti-cancer agents such as microtubule targeting agents (MTAs) are known to interact with tubulin in at least four binding sites: the colchicine, laulimalide, taxane/epothilone and vinca alkaloid sites (Kaur et al., 2014). Clinically used MTAs are divided into two classes: microtubule-destabilizing agents and microtubule-stabilizing agents. Microtubule-destabilizing agents such as vincristine and vinblastine are FDA approved for treating tumors such as Hodgkin lymphoma, whereas microtubule-stabilizing agents such as paclitaxel and docetaxel are used in diverse solid tumors including breast, non-small cell lung, ovarian, and prostate cancer (Dumontet and Jordan, 2010; Kaur et al., 2014). These agents lead to cell cycle arrest in G2/M phase and in turn induce cancer cell apoptosis. Combination therapy is a strategy to treat highly metastatic, advanced malignant or MTA resistant cancers (Lu et al., 2012). It has been reported that aurora kinase inhibitors combined with MTAs can be used to treat human metastatic breast cancer and multidrug-resistant TNBC cells (Bush et al., 2013). MTAs combined with Hsp90 inhibitors can enhance MTAs-induced cell death in preclinical models of non-small cell lung cancer (O’Connell et al., 2014). Farnesyl transferase (FT) inhibitors incorporated with paclitaxel can enhance HDAC6-dependent tubulin deacetylation in breast and lung carcinoma cells (Marcus et al., 2005). Moreover, medicinal chemists have developed strategies to synthesize compounds with dual inhibition effects (Lee et al., 2017). For instance, Guerrant et al. (2012) have manufactured dual HDAC/Topo II inhibitors that induce variant cancer cell growth suppression.

We have previously synthesized a novel 1-benzylindole derivative, MPT0B451, and demonstrated that MPT0B451 can inhibit tubulin assembly and HDAC isoforms activities with a 10-fold selectivity for HDAC6 versus other HDACs (Lee et al., 2017). In this study, we found that MPT0B451 can interact with HDAC6 protein via forming hydrogen bonds with residues His610 and His611. Molecular docking analysis revealed that MPT0B451 contains a styrene linker to stabilize the compound within the HDAC6 binding site. Furthermore, MPT0B451 has four methoxy groups that can stabilize its position when it binds to colchicine binding site of tubulin and facilitate microtubule depolymerization. Herein, MPT0B451 induced acetyl-α-tubulin expression in both human prostate cancer cells and leukemic cells via HDAC6 activity inhibition. We also observed that MPT0B451 induced G2/M cell cycle arrest and apoptosis through the activation of pro-apoptotic proteins. Importantly, our xenograft models showed that the MPT0B451 treatment significantly suppressed tumor growth. Collectively, our results demonstrated the dual inhibitory function of MPT0B451 and provided the molecular mechanisms underlying the anti-tumor activity of MPT0B451.

## Materials and Methods

### Chemicals and Antibodies

MPT0B451, a dual HDAC6 and tubulin inhibitor was synthesized by Dr. Jing-Ping Liou (School of Pharmacy, College of Pharmacy, Taipei Medical University, Taiwan). Vincristine, 3-(4,5-dimethylthiazol-2-yl)-2,5-diphenyltetrazolium bromide (MTT) and propidium iodide (PI) were purchased from Sigma Chemical Co. (St. Louis, MO, United States). Primary antibodies against Caspase 8 (06-775), MPM2 (pSer/pThr) (05-368) and GAPDH (AB2302) were purchased from Millipore (Bedford, MA, United States). The antibodies against Acetyl-Histone H3 (Lys9) (#9649), Histone H3 (#9715), Acetyl-α-tubulin (#3971), α-tubulin (#2144), Cyclin B1(#4135), Aurora A (#14475), Aurora A (Thr288) (#3079), PLK1 (#4535), PLK1 (Thr210) (#9062), cleaved caspase-3 (#9664), PARP (#9542), and caspase 9 (#9508) were purchased from Cell Signaling Technology (Beverly, MA, United States). The antibodies against Cdc2 (SC-54) and cdc25c (SC-372) as well as the labeled secondary antibodies goat anti-rabbit IgG-HRP (SC-2004) and goat anti-mouse IgG-HRP (SC-2005) were purchased from Santa Cruz Biotechnology (Santa Cruz, CA, United States).

### Cell Lines

Human acute myeloid leukemia cell line HL-60 and human prostate cancer cell line PC-3 were purchased from American Type Culture Collection (ATCC, Manassas, VA, United States). HL-60 and PC-3 cells were maintained in RPMI-1640 with 10% (*v/v*) inactive fetal bovine serum, penicillin (100 units/mL) and streptomycin (100 μg/mL, Biological Industries Ltd., Kibbutz Beit HaEmek, Israel). All cells were incubated in an incubator in the presence of 5% CO_2_ at 37°C.

### MTT Assay

HL-60 cells were seeded in 24-well plates at a density of 4 × 10^5^ cells/well with 1 mL culture medium and PC-3 cells were seeded in 96-well plates (5,000 cells/well) with 0.1 mL culture medium then treated with different concentrations of MPT0B451 for 24 or 48 h. Cell viability was determined by treating the cells with MTT (0.5 mg/mL in PBS) for 1 h at 37°C. The crystal formazan dyes were then dissolved in 1 mL sodium acetate buffer (0.1 M, for HL-60) (Chao et al., 2015) or 0.1 mL DMSO (for PC-3). The absorbance was spectrophotometrically analyzed at 550 nm by an ELISA reader (Molecular Devices, Sunnyvale, CA, United States).

### Bioinformatics and Protein Modeling

The co-crystallized structures of the tubulin (PDB ID: 4O2B) and HDAC6 (PDB ID: 5EDU) were retrieved from the RCSB PDB (Berman et al., 2000) website^1^ and prepared using LeadIT (LeadIT BiosolveIT, 2011). All potential water molecules were removed from the binding sites. The co-crystallized ligands colchicine and Trichostatin A were selected for determining binding sites of tubulin and HDAC6, respectively. The binding sites included a radius of 12 Å from the co-crystallized ligands. The docking parameters were set to default. Images and residue interactions were analyzed using Pymol (Schrodinger, 2010) and LeadIT.

### Flow Cytometry

The cell cycle as evaluated by flow cytometry. HL-60 cells (2 × 10^6^ cells/well) and PC-3 cells (4 × 10^5^ cells/well) were seeded in 6-well plates in 2 mL culture medium and treated with gradient concentration of MPT0B451 for the indicated periods. After drug treatment, cells were collected, washed with cold PBS and fixed with 70% (v/v) ice cold ethanol at -20°C for 30 min. The fixed cells were centrifuged to remove the ethanol, resuspended in 0.1 mL DNA extraction buffer (0.2 M Na_2_HPO_4_-0.1 M citric buffer, pH 7.8) for 20 min. The cells were centrifuged and stained with 0.5 mL PI staining buffer (80 μL/mL PI, 100 μL/mL RNase A and 1% Triton X-100 in PBS) for 30 min. Cell cycle distribution was analyzed by FACScan Flow cytometer and Cell Quest software (Becton Dickinson, Mountain View, CA, United States).

### Western Blot Analysis

Cells were incubated with various concentrations of MPT0B451 or vincristine, harvested, washed with PBS, lysed in lysis buffer (50 mM Tris pH 7.4, 150 mM NaCl, 1% Triton X-100, 1 mM EDTA, 1 mM EGTA, 1 mM PMSF, 10 μg/mL aprotinin, 10 μg/mL leupeptin, 1 mM sodium orthovanadate, and 1 mM NaF), and then centrifuged for 30 min at 14,000 rpm at 4°C. The harvested total protein was quantified by BCA Protein Assay Kit (Thermo Fisher Scientific, Rockford, IL, United States). Whole cell extracts were mixed with 5× sample buffer (312.5 mM Tris pH 6.8, 10% SDS, 50% glycerol, 0.05% bromophenol blue and 10% 2-mercaptoethanol) at 95°C for 10 min. Equal amount of total protein samples were separated by SDS–PAGE and subsequently transferred onto PVDF membranes. The membranes were blocked with 5% non-fat milk in PBS for 1 h at room temperature, and incubated with primary antibodies in PBST buffer (0.1% Tween 20 in PBS) at 4°C overnight. The membranes were washed with PBST, followed by incubation with the corresponding HRP-conjugated secondary antibodies diluted in 0.5% non-fat milk in PBST for 1 h at room temperature. Bound antibodies were measured using an enhanced chemiluminescence detection kit (Amersham, Buckinghamshire, United Kingdom). The relative intensity of protein expression was calculated by Image J software (National Institutes of Health, United States).

### KINOME*scan* Assay

The protein kinase selectivity of MPT0B451 (1 μM) was detected in a high-throughput binding assay (KINOME*scan*, DiscoveRx, Fremont, CA, United States) against a panel of 97 kinases. For the human kinase dendrogram, red circles indicated the main hits (<5% of control), weak or no hits were labeled with small red circles (10–35% of control) and smaller green circles (>35% of control). TREE*spot* is a proprietary data visualization software developed by KINOME*scan* that shows the protein kinase binding affinity.

### *In Vivo* Xenograft Model

To evaluate the antitumor activity of MPT0B451, 4-week-old male nude mice were subcutaneously injected with 1 × 10^7^ leukemic cells (HL-60) or prostate cancer cells (PC-3). When the tumor sizes reached 200 mm^3^, mice were separated into three treatment groups (six mice in each group). MPT0B451 was applied with indicated dosage (50 or 100 mg/kg) by intraperitoneal injection (ip), once daily (qd). During the experiment, the tumor size and body weight were measured twice each week. Tumor growth inhibition (TGI) was calculated by dividing the tumor volumes from treatment groups by those of the control groups as 100%. Animal experiments were performed in accordance with relevant guidelines and regulations followed ethical standards, and protocols has been reviewed and approved by Animal Use and Management Committee of Taipei Medical University (IACUC number: LAC-2015-0240).

### Data Analysis and Statistics

All experiments were done three times independently with the data presented as mean ± SD and analyzed by Student’s *t-test*. The detail IC_50_ calculation was described in Nevozhay (2014). The animal experiments were determined by the Mann–Whitney test. Parameters with *p*-value < 0.05 are considered statistically significant.

## Results

### MPT0B451 Inhibits HDAC Activity and Cell Proliferation in Human Cancer Cell Lines

**FIGURE 1:**
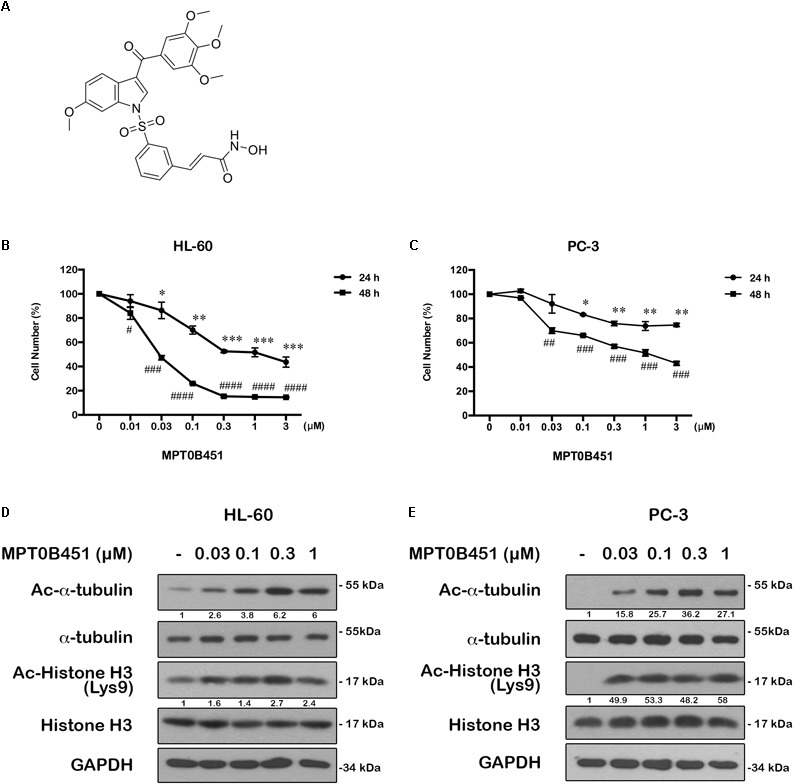
The structure of MPT0B451 and its potential anticancer effect in cancer cell lines. **(A)** Structure of 1-benzylindole derivative compound, MPT0B451. The human cancer cell lines HL-60 **(B)** and PC-3 **(C)** were treated with various concentrations of MPT0B451 for 24 and 48 h, and cell viability was measured by MTT assay. The results represent the mean ± SD of three independent experiments at ^∗^*p* < 0.05, ^∗∗^*p* < 0.01 and ^∗∗∗^*p* < 0.001compared with 24 h control group; ^#^*p* < 0.05, ^##^*p* < 0.01, ^###^*p* < 0.001 and ^####^*p* < 0.0001 compared with 48 h control group. **(D,E)** The effect of α-tubulin and histone H3 acetylation changes in HL-60 and PC-3 cells. Cells were culture with the indicated concentrations of MPT0B451 for 12 h then total cell lysates were detected by western blot analysis. The data were repeated at least three independent experiments.

MPT0B451 was derived from1-benzylindoles (**Figure 1A**). We previously synthesized a series of 1-benzylindole derivatives with dual inhibitory activity for both HDAC and α-tubulin assembly, and MPT0B451 is one of the potent compounds inhibiting cell proliferation in various solid tumor cells, especially for human prostate cancer cells (PC-3, GI_50_ = 33.09 ± 0.97 nM) (Lee et al., 2017). To further investigate the cytotoxic effects of MPT0B451, we found that it can induce cytotoxicity in acute myeloid leukemia cell line, HL-60 (IC_50_ = 42 ± 0.04 nM) (**Figure 1B**) and prostate cancer cell line, PC-3 (IC_50_ = 1.1 ± 0.11 μM) (**Figure 1C**).

MPT0B451 has been shown to inhibit HDAC activity (IC_50_ = 135.45 ± 4.23 nM), particularly HDAC1, 2 (class I) and 6 (class IIb). Notably, MPT0B451 is 12-fold more selective inhibition toward HDAC6 over HDAC2 (Lee et al., 2017). In HL-60 and PC-3 cells, biomarkers of HDAC inhibition, such as acetylated histone H3 and α-tubulin, were increased upon MPT0B451 treatment with a dose-dependent manner (**Figures 1D,E**). Thus, MPT0B451 is an efficient inhibitor against HDAC6 and effectively induces cytotoxicity in human prostate cancer cells and acute myeloid leukemia cells.

### Molecular Docking Analysis Supports That MPT0B451 Is a Dual Effect Inhibitor

**FIGURE 2:**
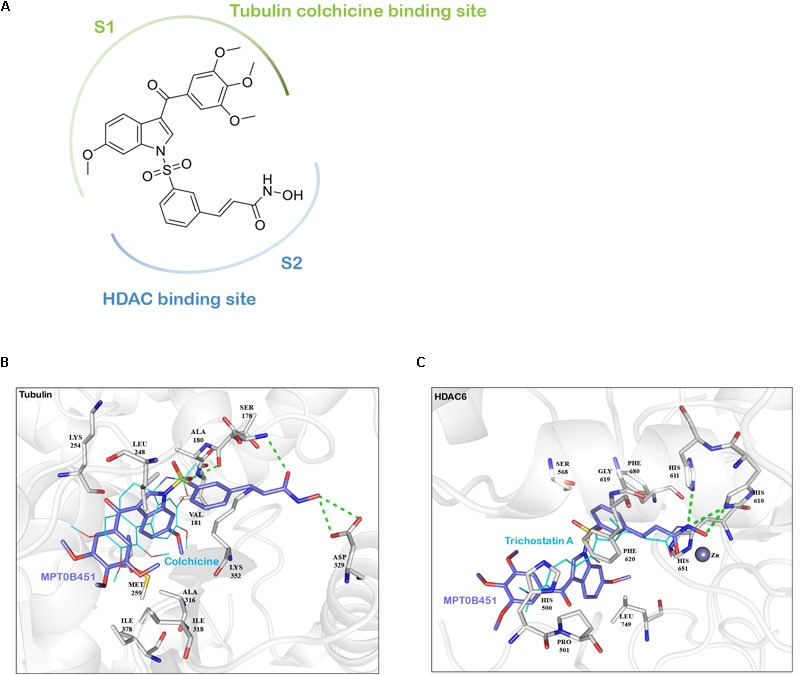
The interaction between MPT0B451 and target proteins analyzes by molecular docking analysis. **(A)** MPT0B451 can be separated into two sub-structures: 6-methoxy-3-(3′,4′,5′-trimethoxybenzoyl)-1H-indole (S1, green) and (E)-N-hydroxy-3-(3-sulfonylphenyl)acrylamide (S2, blue). **(B)** MPT0B451 (purple) is docked into the colchicine binding site of tubulin (gray, PDB ID: 4O2B). The co-crystallized ligand, colchicine (aqua), was used as a reference and aligned to MPT0B451. Green lines represent hydrogen bonds. Residues are labeled as shown. **(C)** MPT0B451 (purple) is docked into HDAC6 (gray, PDB ID: 5EDU). The co-crystallized ligand Trichostatin A (aqua) was used as a reference and aligned to MPT0B451. Green lines represent hydrogen bonds. Residues are labeled as shown.

In addition to inhibiting HDAC6 activity, our previous findings demonstrated that MPT0B451 also inhibits microtubule assembly (Lee et al., 2017). To understand how MPT0B451 acts on HDAC6 and microtubule, we employed molecular docking analysis to examine how MPT0B451 interacts with tubulin and HDAC6. MPT0B451 was docked into the X-ray crystal structure of the colchicine binding site of tubulin (PDB ID: 4O2B) and into the HDAC6 (PDB ID: 5EDU) using LeadIT (LeadIT BiosolveIT, 2011). This compound was designed as a dual inhibitor for HDAC6 and tubulin that consists of two main sub-structures: 6-methoxy-3-(3′,4′,5′-trimethoxybenzoyl)-1H-indole (*S1*) and a (E)-N-hydroxy-3-(3-sulfonylphenyl)acrylamide (*S2*) (**Figure 2A**). We observed that each sub-structure interacts with the tubulin or HDAC6 binding site differently.

The colchicine binding site is potentially important for microtubule depolymerization (Lu et al., 2012). The molecular docking analysis of MPT0B451 in tubulin showed a partial overlap with colchicine, with sub-structure S1 occupying the colchicine binding site (**Figure 2A**). Colchicine contains four methoxy groups that create favorable interactions and stabilize its position within the colchicine binding site (Andreu et al., 1998). Similarly, sub-structure S1 contains two aromatic rings with four methoxy groups (**Figure 2A**), which occupy similar positions as colchicine methoxy groups and are stabilized with hydrophobic interactions by residues Met259, Ala316, Ile318, and Ile378 of tubulin (**Figure 2B**). Instead of occupying the colchicine binding site, sub-structure S2 extends into the binding site and forms two hydrogen bonds with residues Ser178 and Asp329 (**Figure 2B**).

Molecular docking analysis of MPT0B451 and HDAC6 showed common features with traditional HDAC inhibitors (Falkenberg and Johnstone, 2014). Sub-structure S2 contains a N-hydroxyformamide moiety that coordinates with the zinc ion in HDAC6 and forms hydrogen bonds with residues His610 and His611 (**Figure 2C**). These two residues are involved with the catalytic core of HDAC6 and form similar interactions with known HDAC inhibitors Trichostatin A or SAHA (Bertrand, 2010). Sub-structure S2 also contains a styrene linker that stabilizes the compound within the HDAC6 binding site and passes through the hydrophobic channel of the binding site. This creates hydrophobic interactions with residues Ser568, Gly619, Phe620, His651, and Phe680 (**Figure 2C**). Sub-structure S1 does not enter the binding site in HDAC6. Instead, it can be recognized as an inhibitor cap that occludes the entrance of the binding site (**Figure 2C**).

These findings supported the notion that MPT0B451 is a dual inhibitor, which was designed by including two sub-structures, 6-methoxy-3-(3′,4′,5′-trimethoxybenzoyl)-1H-indole (S1) and (E)-N-hydroxy-3-(3-sulfonylphenyl)acrylamide (S2), that exploit key interactions within the β-tubulin and HDAC6 binding sites, respectively (**Figure 2A**).

### Effects of MPT0B451 on Mitotic Arrest in HL-60 and PC-3 Cells

**FIGURE 3:**
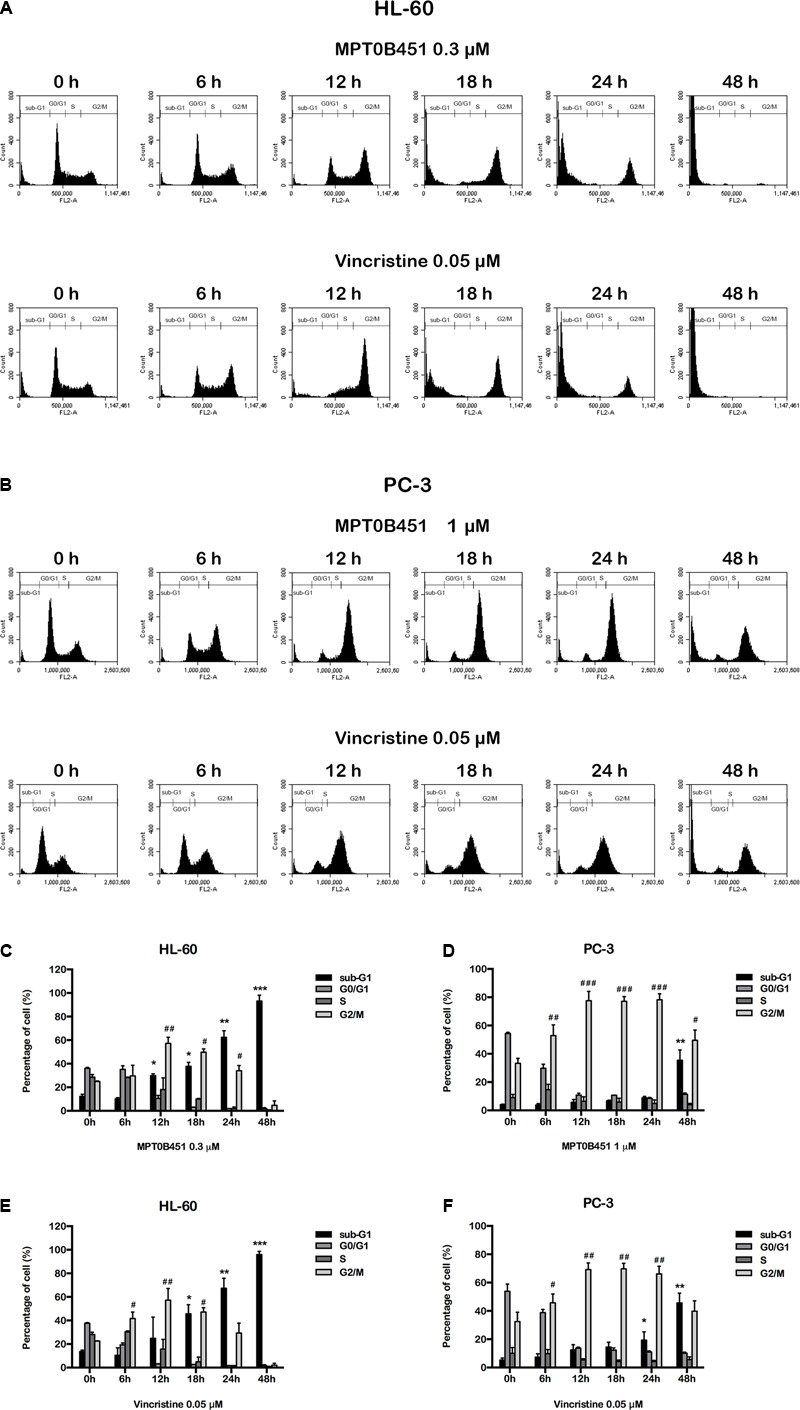
MPT0B451 induces mitotic arrest in HL-60 and PC-3 cells. The histograms of cell cycle distribution were detected by flow cytometry. **(A)** HL-60 cells were treated with MPT0B451 (0.3 μM), and **(B)** PC-3 cells were cultured in 1 μM MPT0B451 for 6, 12, 18, 24, and 48 h. **(C–F)** The statistical analysis of cell cycle distribution. **(C,E)** HL-60 cells were treated with MPT0B451 or vincristine, and **(D,F)** PC-3 cells were treated with MPT0B451 or vincristine for 6, 12, 18, 24, and 48 h. The data were repeated at least three independent experiments. ^∗^*p* < 0.05, ^∗∗^*p* < 0.01, and ^∗∗∗^*p* < 0.001, compared with sub-G1 0 h untreated group; ^#^*p* < 0.05, ^##^*p* < 0.01, and ^###^*p* < 0.001 compared with G2/M 0 h untreated group.

**FIGURE 4:**
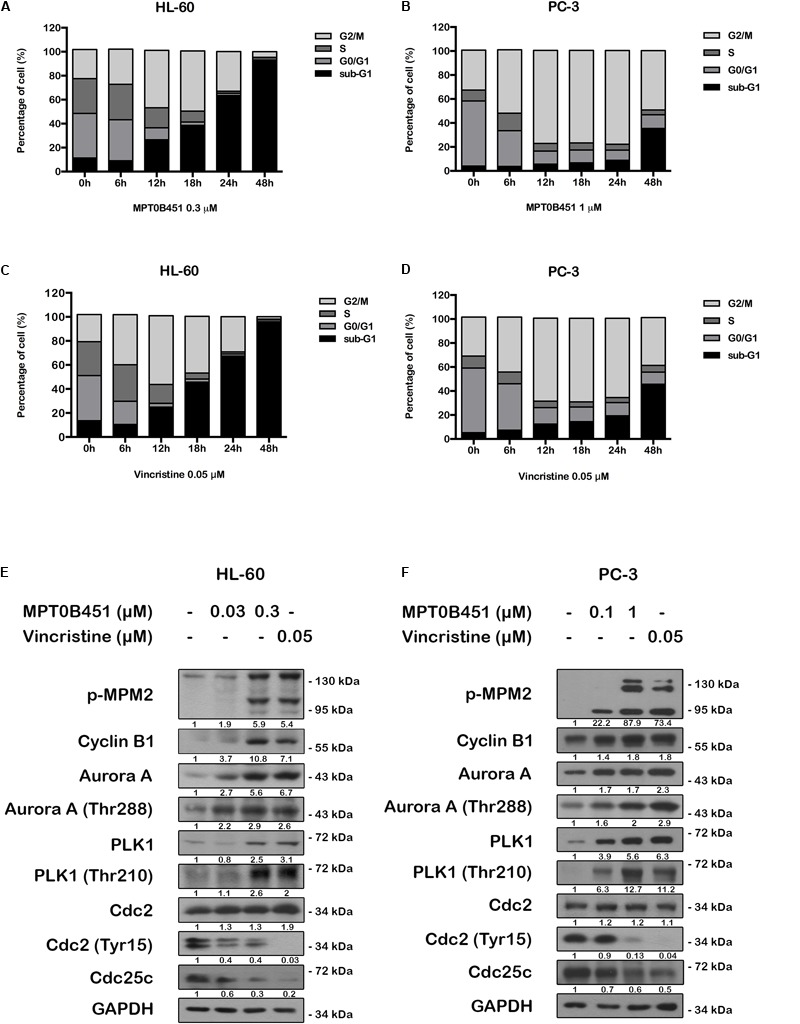
MPT0B451 induces mitotic arrest and alters G2/M proteins transition. The cell cycle was measured by flow cytometry. HL-60 **(A,C)** and PC-3 **(B,D)** cells were incubated with MPT0B451 (HL-60, 0.3 μM and PC-3, 1 μM) or vincristine (0.05 μM) for 6, 12, 18, 24, and 48 h. The quantitative data were showed in time-course. Data mean ± SD of three independent experiments. **(E,F)** MPT0B451 altered proteins levels that regulate the G_2_/M transition. Cells were treated with MPT0B451 (HL-60, 0.03, 0.3 μM and PC-3, 0.1, 1 μM) or vincristine (vin., 0.05 μM) for 12 h to evaluate G_2_/M phase regulatory proteins expression through western blotting. The data were repeated at least three independent experiments.

Since HDAC6 and α-tubulin play important roles in cell cycle regulation (Nowak and Corces, 2004), we investigated the effect of MPT0B451 on cell cycle using HL-60 and PC-3 cells. Twelve to eighteen hours after MPT0B451 treatment, HL-60 cells arrested in G2/M phase (**Figures 3A,C, 4A**). Similar result was observed in PC-3 cells after 6–24 h treatment of MPT0B451 (**Figures 3B,D, 4B**). Importantly, the effect of MPT0B451 on G2/M arrest was similar to a chemotherapeutic agent, vincristine, which induced mitotic arrest via microtubule depolymerization (**Figures 3E,F, 4C,D**; Lee et al., 2017).

We further evaluated the level of G2/M regulatory proteins using western blotting. The expression of mitosis-specific phosphorylated MPM2, cyclin B1, aurora kinase A (Aurora A), p-Thr288 Aurora A, serine/threonine kinase polo-like kinase 1 (PLK1) and p-Thr210 PLK1 were increased, and p-Tyr15 Cdc2 and Cdc25c were decreased in both HL-60 and PC-3 cells after 12 h of MPT0B451 treatment (**Figures 4E,F**). These results showed that MPT0B451 modulates G2/M transition proteins and promotes G2/M arrest.

### MPT0B451 Induces Cell Apoptosis

**FIGURE 5:**
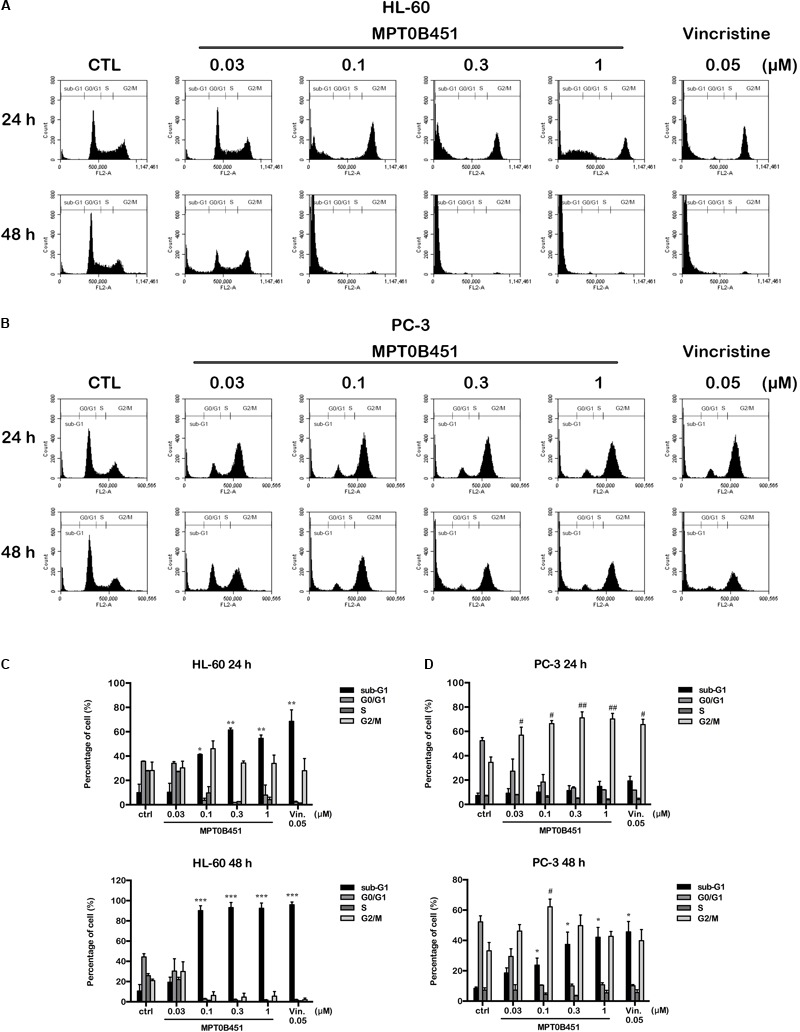
The kinetic changes of cell cycle under MPT0B451 treatment. The histograms of cell cycle distribution were detected by flow cytometry. **(A)** HL-60 and **(B)** PC-3 cells were treated in indication concentrations of MPT0B451 (0.03, 0.1, 0.3, and 1 μM) or vincristine 0.05 μM for 24 and 48 h, respectively. The data were expressed at least three separate determinations. The statistical data of cell cycle distribution. **(C)** HL-60 and **(D)** PC-3 were cultured in MPT0B451 or vincristine for 24 and 48 h. The data were repeated at least three independent experiments. ^∗^*p* < 0.05, ^∗∗^*p* < 0.01 and ^∗∗∗^*p* < 0.001 compared with sub-G1 untreated group; ^#^*p* < 0.05 and ^##^*p* < 0.01 compared with G2/M untreated group.

**FIGURE 6:**
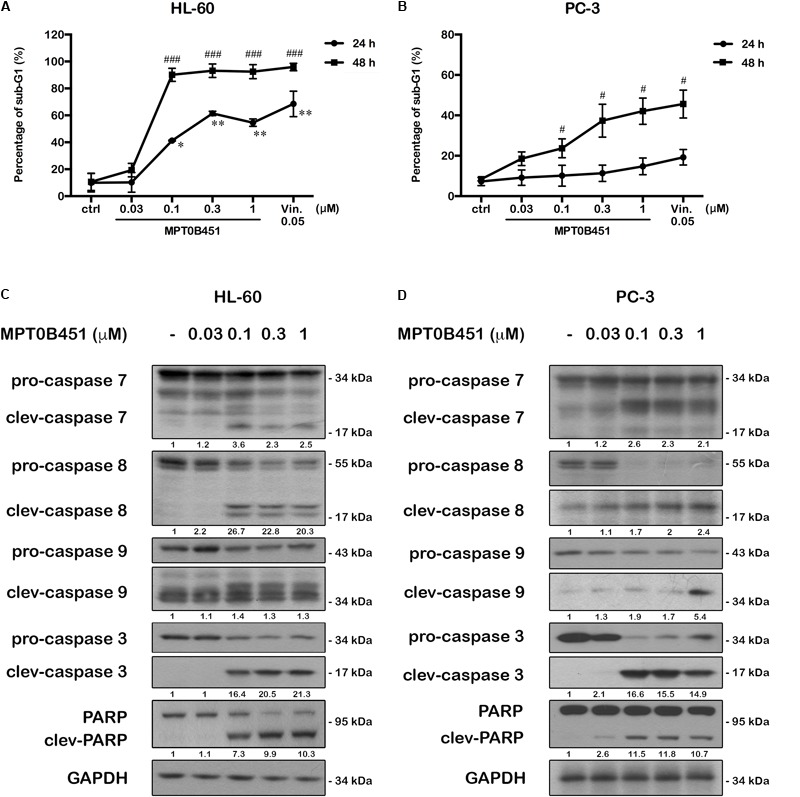
MPT0B451 induces cell apoptosis and activates apoptotic protein expression in HL-60 and PC-3 cells. The sub-G1 profiles of HL-60 **(A)** and PC-3 **(B)** cells were treated with indicated concentrations of MPT0B451 or vincristine (vin., 0.05 μM) for 24 or 48 h, and detected by flow cytometry. The results represent the mean ± SD of three independent experiments at ^∗^*p* < 0.05 and ^∗∗^*p* < 0.01 compared with 24 h control group; ^#^*p* < 0.05 and ^###^*p* < 0.001 compared with 48 h control group. **(C,D)** Apoptotic proteins were activated after MPT0B451 treatment for 24 h (HL-60) or 48 h (PC-3). The whole cell lysates were subjected to western blotting, and the data were repeated at least three independent experiments.

Forty-eight hours after MPT0B451 or vincristine treatment, an accumulation of sub-G1 cells was observed in both HL-60 and PC-3 cell lines (**Figures 4A–D**). The representative histograms of cell cycle distribution and the statistical data were showed in **Figure 5**. The sub-G1 population of HL-60 cells was increased with elevated MPT0B451 concentration after 24 and 48 h of treatment (**Figures 5A, 6A**), whereas the sub-G1 population of PC-3 cells increased dramatically only after 48 h treatment (**Figures 5B, 6B**). Similar to vincristine treatment, the sub-G1 DNA content increased significantly in PC-3 and HL-60 cells treated with MPT0B451 indicating cells are undergoing apoptosis. We therefore investigated the level of apoptotic related proteins. Western blotting analysis showed that MPT0B451 treatment resulted in decreases in pro-caspase 3, 8 and 9 and increases in cleaved forms of caspase 3, 7, 8, 9 and poly (ADP-ribose) polymerase (PARP) (**Figures 6C,D**) indicating that MPT0B451 can activate apoptotic pathway in both HL-60 and PC-3 cancer cells despite of with different efficiency.

### KINOME*scan* Analysis Did Not Detect Significant Kinase Inhibition Activity in MPT0B451

**FIGURE 7:**
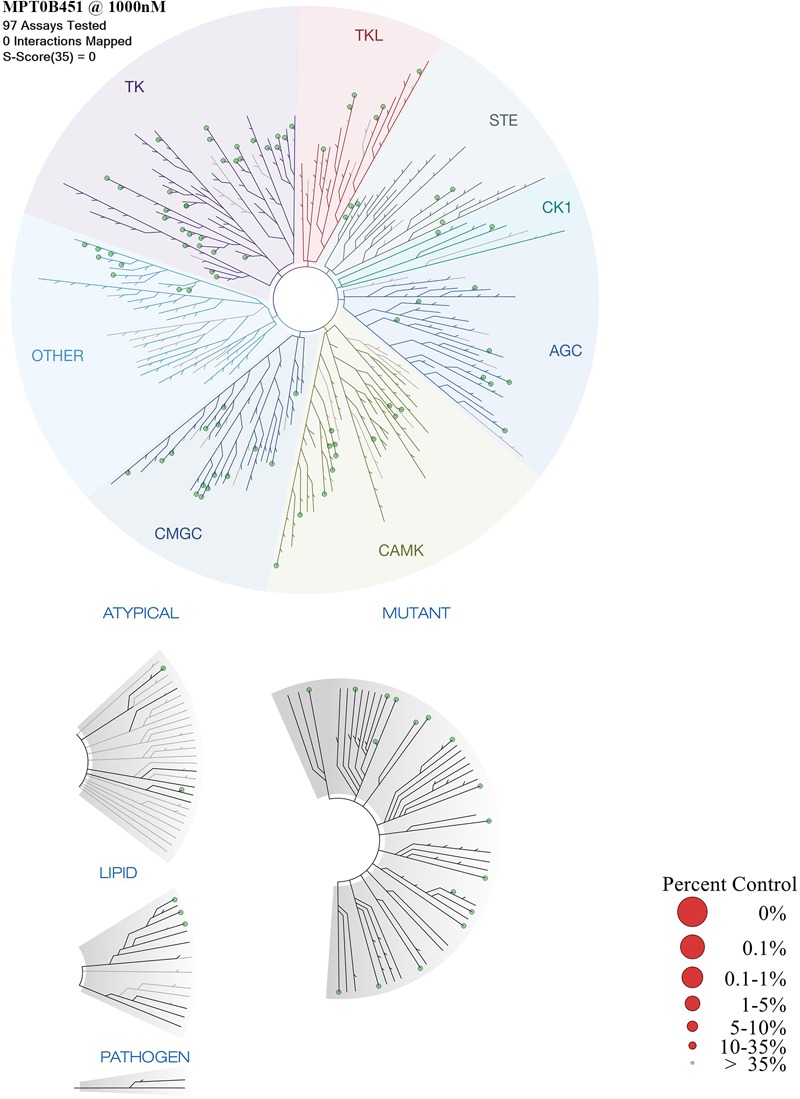
TREE*spot*^TM^ interaction maps for MPT0B451 detected by proprietary active site-directed screening KINOME*scan*. The kinase binding affinity of MPT0B451 (1 μM) was detected by a high-throughput binding assay (KINOME*scan*, DiscoveRx). For the human kinase dendrogram, red circles mean the main hits (<5% of control), weak or no hits are labeled with small red circles (10–35% of control) and smaller green circles (>35% of control).

Previous data demonstrated that MPT0B451 inhibits HDAC6 activity and modulates microtubule assembly (Lee et al., 2017). In order to confirm that MPT0B451 is specifically target HDAC6 and microtubule but not other kinases, we conduct kinase profiling using DiscoveRx KINOME*scan* technology (97 protein kinases). For the human kinase dendrogram, red circles mean the main hits (<5% of control), weak or no hits are labeled with small red circles (10–35% of control) and smaller green circles (>35% of control). The human kinase dendrogram revealed that all assayed kinases still showed more than 60% of enzymatic activities with the presence of MPT0B451 compared to the control (**Figure 7** and **Table 1**) indicating that MPT0B451 does not exhibit kinase inhibition activity toward the kinases being tested. Therefore, MPT0B451 is an effective dual inhibitor by targeting both tubulin and HDAC6.

**Table 1:** Matrix of MPT0B451 screens of KINOME*scan*.

Target	MPT0B451	Target	MPT0B451
Gene symbol	%Ctrl @ 1000 nM	Gene symbol	%Ctrl @ 1000 nM
ABL1(E255K)-phosphorylated	81	KIT(D816V)	100
ABL1(T315I)-phosphorylated	92	KIT(V559D,T670I)	96
ABL1-nonphosphorylated	86	LKB1	100
ABL1-phosphorylated	93	MAP3K4	100
ACVR1B	90	MAPKAPK2	85
ADCK3	99	MARK3	100
AKT1	88	MEK1	100
AKT2	88	MEK2	98
ALK	100	MET	100
AURKA	91	MKNK1	100
AURKB	99	MKNK2	76
AXL	86	MLK1	97
BMPR2	99	p38-alpha	91
BRAF	99	p38-beta	83
BRAF(V600E)	100	PAK1	100
BTK	100	PAK2	100
CDK11	69	PAK4	97
CDK2	100	PCTK1	90
CDK3	100	PDGFRA	100
CDK7	72	PDGFRB	100
CDK9	100	PDPK1	67
CHEK1	97	PIK3C2B	91
CSF1R	100	PIK3CA	100
CSNK1D	98	PIK3CG	94
CSNK1G2	96	PIM1	73
DCAMKL1	83	PIM2	97
DYRK1B	85	PIM3	100
EGFR	100	PKAC-alpha	99
EGFR(L858R)	97	PLK1	100
EPHA2	98	PLK3	79
ERBB2	90	PLK4	91
ERBB4	79	PRKCE	96
ERK1	94	RAF1	84
FAK	100	RET	92
FGFR2	94	RIOK2	100
FGFR3	100	ROCK2	100
FLT3	100	RSK2(Kin.Dom.1-N-terminal)	99
GSK3B	100	SNARK	95
IGF1R	100	SRC	100
IKK-alpha	100	SRPK3	77
IKK-beta	100	TGFBR1	94
INSR	98	TIE2	76
JAK2(JH1domain-catalytic)	100	TRKA	96
JAK3(JH1domain-catalytic)	75	TSSK1B	90
JNK1	81	TYK2(JH1domain-catalytic)	99
JNK2	97	ULK2	100
JNK3	97	VEGFR2	95
KIT	99	YANK3	96
		ZAP70	99

*The kinase targets and the remaining activity (percent of control, POC) by MPT0B451.*

### Xenograft Models Demonstrate the Anti-tumor Effect of MPT0B451

**FIGURE 8:**
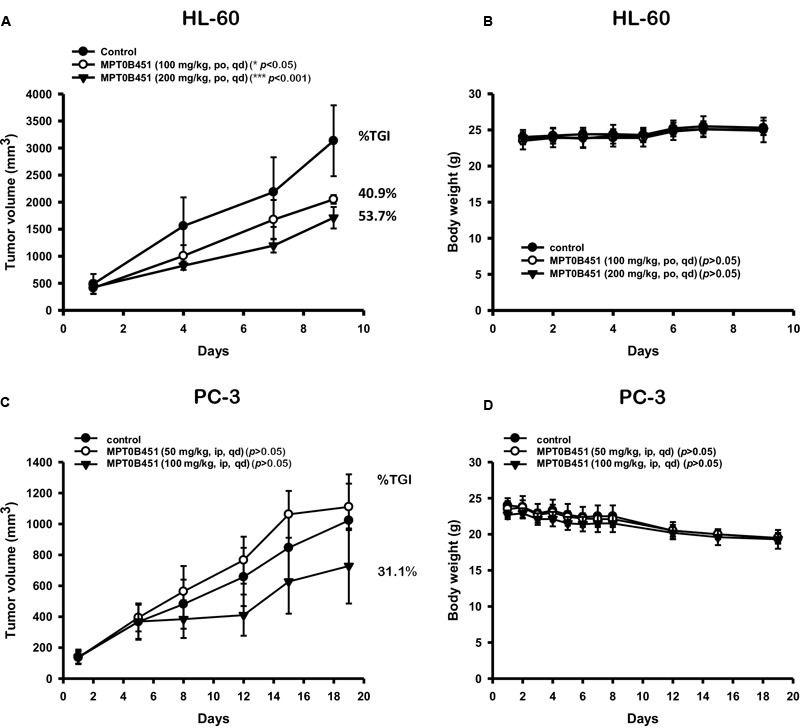
Anti-tumor activity of MPT0B451 in HL-60 and PC-3 xenograft models. Male nude mice subcutaneously injected of HL-60 **(A)** or PC-3 **(C)** cells were divided into three groups (*n* = 6), and received with indicated doses of MPT0B451 by intraperitoneal injection (ip), once daily (qd). The tumor volume and the body weight **(B,D)** were expressed as mean ± SD, and the percentage of TGI was determined.

To further evaluate the anti-tumor activity of MPT0B451, we subcutaneously injected human leukemia cells (HL-60) or human prostate cancer cells (PC-3) in mouse xenograft models. Once tumors were palpable (approximately 200 mm^3^), mice were randomized into control (vehicle) and treatment groups (*n* = 6/group). We found that MPT0B451 suppressed the growths of tumors derived from HL-60 and PC-3 cells in dose-dependent manners compared to the control treatment. The percentage of TGI were 40.9 and 31.1% in HL-60 and PC-3 grafted mice, respectively (**Figures 8A,C**). None of the treated mice showed significant body weight loss (**Figures 8B,D**). Together, our cell-based observations and animal models both demonstrated that MPT0B451 exhibits anti-tumor activity in human leukemia cells and prostate cancer cells. Such activity may be resulted from the dual HDAC6 and anti-mitotic effect of MPT0B451 to induce G2/M arrest and apoptosis.

## Discussion

Cytotoxic drugs and MTAs are common therapeutic treatments for prostate cancer and acute leukemia. However, neuronal toxicity, drug resistance, and deteriorating patients continues to plague patients. The development of a multi-target drug may overcome these side effects. HDAC inhibitors have been verified for cancer cell growth inhibition, cell apoptosis inducement, cell invasion, and metastasis suppression (Amengual et al., 2015, 2016). Currently, four HDAC inhibitors are approved by the FDA for T-cell lymphoma and multiple myeloma (Kawaguchi et al., 2003; Aldana-Masangkay and Sakamoto, 2011; Liu et al., 2016). Furthermore, a selective HDAC6 inhibitor with a proteasome inhibitor has been employed in multiple myeloma (Aldana-Masangkay and Sakamoto, 2011; Santo et al., 2012). Various studies have indicated that combining a pan-HDAC or HDAC6 inhibitor with other anti-cancer drugs may increase efficacy (Cosenza et al., 2017). As the result of other studies, HDAC6 has highly expressed in various cancer types including leukemia, prostate carcinoma, colorectal carcinoma and breast cancer compared with normal tissue, and the results also confirmed the high level of HDAC6 in HL-60 and PC-3 cells (Parmigiani et al., 2008; Hackanson et al., 2012). Above these finding, developing compounds that also target a different mechanism is a viable strategy to create a synergistic effect for cancer treatment. In this study, we showed that MPT0B451, a novel 1-benzylindole derivative inhibitor, selectively inhibits HDAC6 activity and microtubule assembly to facilitate the efficacy in anti-cancer treatment.

Multi-target drugs have the potential to increase therapeutic effects by aiming multiple targets as well as overcome drug resistance that arise from mutations (Csermely et al., 2005; Boran and Iyengar, 2010; Talevi, 2015). To design such an inhibitor, we synthesized a multi-target compound that targets both the colchicine-tubulin and HDAC6 binding sites. Our previous study indicated that MPT0B451 is a selective inhibitor of HDAC6 (Lee et al., 2017). In this study, we showed that MPT0B451 significantly increased the acetylation of histone H3 and tubulin (**Figures 1D,E**). The structure of MPT0B451 is composed of two sub-structures (**Figure 2**). The tubulin specific structure S1 occupies the same space as colchicine. Both MPT0B451 and colchicine contain four methoxy groups that occupy similar positions. Previous studies have suggested that the methoxy groups create high affinity for the colchicine binding site (Bhattacharyya et al., 2008; Sapra et al., 2013). MPT0B451 is able to exploit the colchicine binding site with similar interactions observed in tubulin inhibitors (Lu et al., 2012). Importantly, the HDAC6 specific structure does not negatively interfere with the colchicine binding site. The molecular analysis revealed that MPT0B451 contains three essential components seen in traditional HDAC inhibitors: a zinc binding group, linker, and a cap region (Falkenberg and Johnstone, 2014). The zinc binding group is critical for HDAC inhibitor recognition (Dowling et al., 2008). The sub-structure S2 contains the zinc binding group N-hydroxyformamide and a styrene linker, which are also found on panobinostat (Atadja, 2009). The S1 sub-structure acts as an inhibitor cap and does not hinder the HDAC6 binding (**Figure 2**). Additionally, KINOME*scan* data demonstrated that MPT0B451 may not exhibit kinase inhibition activity (**Figure 7** and **Table 1**). Therefore, it is likely that MPT0B451 induces mitotic arrest and activates apoptotic pathway (**Figures 3–6**) specifically through HDAC6 and tubulin inhibition but not via other kinase pathway. If a drug hits multiple targets, for instance other kinases, it might increase the potential risk of side effects. Previous studies in sorafenib showed that it is a multi-targeted kinase inhibitor. Besides inhibits c-Raf and b-Raf kinases, it also potentially inhibits VEGFR-2, VEGFR-3, Fit-3, c-Kit, and PDGFR (Widakowich et al., 2007). However, VEGFR-2 inhibition is related to a skin toxicity known as hand/foot skin rash (HFSR) (Jain et al., 2010), as a result, patients under sorafenib treatment are commonly develop skin toxicity (Widakowich et al., 2007; Senapati et al., 2014). Thus, KINOME*scan* analysis demonstrated that MPT0B451 did not have kinase inhibition ability may have less risks in side effect without other potential targets.

Previous research also found that HDAC inhibitors associate with microtubule dynamics, such as acetylation and polymerization, via HDAC6 inhibition (Kawaguchi et al., 2003; Zhang et al., 2003; Aldana-Masangkay and Sakamoto, 2011; Simoes-Pires et al., 2017). We showed that the cell cycle is evidently arrested at G2/M phase in HL-60 and PC-3 cells upon treatment of MPT0B451 (**Figures 3A,B, 4A,B**), and the expression of G2/M transition regulatory proteins confirmed the cell arrest. Cdc2 is inactivated when Cdc25c, a tyrosine phosphatase, removes the phosphorylation at Tyr15. In contrast, phosphorylation of Thr161 contributes to Cdc2 activation and results in the formulation of a Cdc2/Cyclin B1 complex to facilitate cell transition to the M phase (Castedo et al., 2004; Nowak and Corces, 2004). Our data indicated that the expression of p-Tyr15 Cdc2 was decreased and the levels of cyclin B1, aurora A, PLK1 and p-Thr210 PLK1 were increased. Additionally, the M phase marker, mitosis-specific phosphorylated MPM2, was upregulated under MPT0B451 treatment (**Figures 4E,F**). These data suggest that MPT0B451 induces cell cycle arrest in M phase but not in G2 phase. Moreover, the cell cycle distribution also showed that the population of sub-G1 phase was increased (**Figures 6A,B**), and induced apoptotic proteins activation, such as cleavage caspase 3, 7, 8, 9 and PARP (**Figures 6C,D**). This is consistent with previous studies describing cell apoptosis induced by HDAC inhibitors (Huang et al., 2015; Chen et al., 2016).

According to molecular docking analysis, MPT0B451 showed partial overlap with colchicine which is an important site for potential microtubule depolymerization (Lu et al., 2012). To date, although colchicine binding site agents have not yet approved as anti-cancer drugs, there are some colchicine binding site inhibitors in clinical trial, such as CA-4P, CA-1P, ABT-751, and CYT997 on solid tumors or malignant hematology (Ji et al., 2015; Nepali et al., 2016; Wang et al., 2016). We developed a compound, MPT0B451, with colchicine binding site and also demonstrated its activity in microtubule depolymerization (Lee et al., 2017). Herein, our data showed the cytotoxic effect both in human acute myeloid leukemia cell HL-60 and pancreatic cancer cell PC-3. It would be of interest to optimize compound MPT0B451 for its inhibition selectivity in HDAC6 and validate the anti-cancer effect in cancer cell lines or patient derived cells resistant to MTAs.

## Conclusion

Our results showed that MPT0B451 not only inhibited HDAC6 activity, but also interfere with microtubule assembly (Lee et al., 2017). This dual inhibition activity may lead to cell cycle arrest in the M phase by activating the M phase marker, phosphorylated MPM2, and trigger cell apoptosis via caspase-dependent pathway in human prostate cancer and leukemia cells. Importantly, MPT0B451 effectively inhibited tumor growth in HL-60 and PC-3 cells grafted mice. These findings suggest that MPT0B451 has potential to be further developed for anti-cancer treatment.

## Author Contributions

Y-WW performed majority of the experiments. T-CH, K-CH, and TL was responsible for preparing the materials and docking. J-PL provided the compound, MPT0B451. Y-LC and S-LP designed the animal study. WH-V, S-LP, and W-CHF organized the manuscript. All authors approved the final manuscript.

## Conflict of Interest Statement

The authors declare that the research was conducted in the absence of any commercial or financial relationships that could be construed as a potential conflict of interest.

## Footnotes

1 http://www.rcsb.org/pdb

## References

[B1] Aldana-Masangkay G. I. Sakamoto K. M. (2011). The role of HDAC6 in cancer. *J. Biomed. Biotechnol.* 2011:875824. 10.1155/2011/875824 21076528 PMC2975074

[B2] Amengual J. E. Johannet P. Lombardo M. Zullo K. Hoehn D. Bhagat G. (2015). Dual targeting of protein degradation pathways with the selective HDAC6 inhibitor ACY-1215 and bortezomib is synergistic in lymphoma. *Clin. Cancer Res.* 21 4663–4675. 10.1158/1078-0432.ccr-14-3068 26116270 PMC4609274

[B3] Amengual J. E. Prabhu S. A. Lombardo M. Zullo K. Johannet P. M. Gonzalez Y. (2016). Mechanisms of acquired drug resistance to the HDAC6 selective inhibitor ricolinostat reveals rational drug-drug combination with ibrutinib. *Clin. Cancer Res.* 23 3084–3096. 10.1158/1078-0432.ccr-16-2022 27993968 PMC5474138

[B4] Andreu J. M. Perez-Ramirez B. Gorbunoff M. J. Ayala D. Timasheff S. N. (1998). Role of the colchicine ring A and its methoxy groups in the binding to tubulin and microtubule inhibition. *Biochemistry* 37 8356–8368. 10.1021/bi9728553 9622487

[B5] Atadja P. (2009). Development of the pan-DAC inhibitor panobinostat (LBH589): successes and challenges. *Cancer Lett.* 280 233–241. 10.1016/j.canlet.2009.02.019 19344997

[B6] Berman H. M. Westbrook J. Feng Z. Gilliland G. Bhat T. N. Weissig H. (2000). The protein data bank. *Nucleic Acids Res.* 28 235–242. 10.1093/nar/28.1.235 10592235 PMC102472

[B7] Bertrand P. (2010). Inside HDAC with HDAC inhibitors. *Eur. J. Med. Chem.* 45 2095–2116. 10.1016/j.ejmech.2010.02.030 20223566

[B8] Bhattacharyya B. Panda D. Gupta S. Banerjee M. (2008). Anti-mitotic activity of colchicine and the structural basis for its interaction with tubulin. *Med. Res. Rev.* 28 155–183. 10.1002/med.20097 17464966

[B9] Boran A. D. W. Iyengar R. (2010). Systems approaches to polypharmacology and drug discovery. *Curr. Opin. Drug Discov. Dev.* 13 297–309. PMC3068535 20443163

[B10] Boyault C. Sadoul K. Pabion M. Khochbin S. (2007). HDAC6, at the crossroads between cytoskeleton and cell signaling by acetylation and ubiquitination. *Oncogene* 26 5468–5476. 10.1038/sj.onc.1210614 17694087

[B11] Bush T. L. Payton M. Heller S. Chung G. Hanestad K. Rottman J. B. (2013). AMG 900, a small-molecule inhibitor of aurora kinases, potentiates the activity of microtubule-targeting agents in human metastatic breast cancer models. *Mol. Cancer Ther.* 12 2356–2366. 10.1158/1535-7163.mct-12-1178 23990115

[B12] Castedo M. Perfettini J. L. Roumier T. Andreau K. Medema R. Kroemer G. (2004). Cell death by mitotic catastrophe: a molecular definition. *Oncogene* 23 2825–2837. 10.1038/sj.onc.1207528 15077146

[B13] Chao M. W. Lai M. J. Liou J. P. Chang Y. L. Wang J. C. Pan S. L. (2015). The synergic effect of vincristine and vorinostat in leukemia *in vitro* and *in vivo*. *J. Hematol. Oncol.* 8:82. 10.1186/s13045-015-0176-7 26156322 PMC4504084

[B14] Chen M. C. Huang H. H. Lai C. Y. Lin Y. J. Liou J. P. Lai M. J. (2016). Novel histone deacetylase inhibitor MPT0G009 induces cell apoptosis and synergistic anticancer activity with tumor necrosis factor-related apoptosis-inducing ligand against human hepatocellular carcinoma. *Oncotarget* 7 402–417. 10.18632/oncotarget.6352 26587975 PMC4808007

[B15] Cosenza M. Civallero M. Marcheselli L. Sacchi S. Pozzi S. (2017). Ricolinostat, a selective HDAC6 inhibitor, shows anti-lymphoma cell activity alone and in combination with bendamustine. *Apoptosis* 22 827–840. 10.1007/s10495-017-1364-4 28315173 PMC5401712

[B16] Csermely P. Agoston V. Pongor S. (2005). The efficiency of multi-target drugs: the network approach might help drug design. *Trends Pharmacol. Sci.* 26 178–182. 10.1016/j.tips.2005.02.007 15808341

[B17] Dowling D. P. Gantt S. L. Gattis S. G. Fierke C. A. Christianson D. W. (2008). Structural studies of human histone deacetylase 8 and its site-specific variants complexed with substrate and inhibitors. *Biochemistry* 47 13554–13563. 10.1021/bi801610c 19053282 PMC2635894

[B18] Dumontet C. Jordan M. A. (2010). Microtubule-binding agents: a dynamic field of cancer therapeutics. *Nat. Rev. Drug Discov.* 9 790–803. 10.1038/nrd3253 20885410 PMC3194401

[B19] Falkenberg K. J. Johnstone R. W. (2014). Histone deacetylases and their inhibitors in cancer, neurological diseases and immune disorders. *Nat. Rev. Drug Discov.* 13 673–691. 10.1038/nrd4360 25131830

[B20] Guerrant W. Patil V. Canzoneri J. C. Oyelere A. K. (2012). Dual targeting of histone deacetylase and topoisomerase II with novel bifunctional inhibitors. *J. Med. Chem.* 55 1465–1477. 10.1021/jm200799p 22260166 PMC3306125

[B21] Hackanson B. Rimmele L. Benkisser M. Abdelkarim M. Fliegauf M. Jung M. (2012). HDAC6 as a target for antileukemic drugs in acute myeloid leukemia. *Leuk. Res.* 36 1055–1062. 10.1016/j.leukres.2012.02.026 22464548

[B22] Huang Y. C. Huang F. I. Mehndiratta S. Lai S. C. Liou J. P. Yang C. R. (2015). Anticancer activity of MPT0G157, a derivative of indolylbenzenesulfonamide, inhibits tumor growth and angiogenesis. *Oncotarget* 6 18590–18601. 10.18632/oncotarget.4068 26087180 PMC4621912

[B23] Hubbert C. Guardiola A. Shao R. Kawaguchi Y. Ito A. Nixon A. (2002). HDAC6 is a microtubule-associated deacetylase. *Nature* 417 455–458. 10.1038/417455a 12024216

[B24] Jain L. Sissung T. M. Danesi R. Kohn E. C. Dahut W. L. Kummar S. (2010). Hypertension and hand-foot skin reactions related to VEGFR2 genotype and improved clinical outcome following bevacizumab and sorafenib. *J. Exp. Clin. Cancer Res.* 29:95. 10.1186/1756-9966-29-95 20630084 PMC2913951

[B25] Ji Y. T. Liu Y. N. Liu Z. P. (2015). Tubulin colchicine binding site inhibitors as vascular disrupting agents in clinical developments. *Curr. Med. Chem.* 22 1348–1360. 25620094 10.2174/0929867322666150114163732

[B26] Kaur R. Kaur G. Gill R. K. Soni R. Bariwal J. (2014). Recent developments in tubulin polymerization inhibitors: an overview. *Eur. J. Med. Chem.* 87 89–124. 10.1016/j.ejmech.2014.09.051 25240869

[B27] Kawaguchi Y. Kovacs J. J. McLaurin A. Vance J. M. Ito A. Yao T. P. (2003). The deacetylase HDAC6 regulates aggresome formation and cell viability in response to misfolded protein stress. *Cell* 115 727–738. 14675537 10.1016/s0092-8674(03)00939-5

[B28] LeadIT BiosolveIT (2011). *LeadIT BiosolveIT.* Available at: http://www.biosolveit.de/LeadIT [accessed March 12].

[B29] Lee H. Y. Lee J. F. Kumar S. Wu Y. W. HuangFu W. C. Lai M. J. (2017). 3-Aroylindoles display antitumor activity in vitro and in vivo: effects of N1-substituents on biological activity. *Eur. J. Med. Chem.* 125 1268–1278. 10.1016/j.ejmech.2016.11.033 27886544

[B30] Li Y. Shin D. Kwon S. H. (2013). Histone deacetylase 6 plays a role as a distinct regulator of diverse cellular processes. *FEBS J.* 280 775–793. 10.1111/febs.12079 23181831

[B31] Liu Y. Li L. Min J. (2016). Structural biology: HDAC6 finally crystal clear. *Nat. Chem. Biol.* 12 660–661. 10.1038/nchembio.2158 27538024

[B32] Lu Y. Chen J. Xiao M. Li W. Miller D. D. (2012). An overview of tubulin inhibitors that interact with the colchicine binding site. *Pharm. Res.* 29 2943–2971. 10.1007/s11095-012-0828-z 22814904 PMC3667160

[B33] Marcus A. I. Zhou J. O’Brate A. Hamel E. Wong J. Nivens M. (2005). The synergistic combination of the farnesyl transferase inhibitor lonafarnib and paclitaxel enhances tubulin acetylation and requires a functional tubulin deacetylase. *Cancer Res.* 65 3883–3893. 10.1158/0008-5472.can-04-3757 15867388 PMC1861827

[B34] Nanduri P. Hao R. Fitzpatrick T. Yao T. P. (2015). Chaperone-mediated 26S proteasome remodeling facilitates free K63 ubiquitin chain production and aggresome clearance. *J. Biol. Chem.* 290 9455–9464. 10.1074/jbc.M114.627950 25713068 PMC4392251

[B35] Nepali K. Ojha R. Lee H. Y. Liou J. P. (2016). Early investigational tubulin inhibitors as novel cancer therapeutics. *Expert Opin. Investig. Drugs* 25 917–936. 10.1080/13543784.2016.1189901 27186892

[B36] Nevozhay D. (2014). Cheburator software for automatically calculating drug inhibitory concentrations from *in vitro* screening assays. *PLoS One* 9:e106186. 10.1371/journal.pone.0106186 25184280 PMC4153570

[B37] Nowak S. J. Corces V. G. (2004). Phosphorylation of histone H3: a balancing act between chromosome condensation and transcriptional activation. *Trends Genet.* 20 214–220. 10.1016/j.tig.2004.02.007 15041176

[B38] O’Connell B. C. O’Callaghan K. Tillotson B. Douglas M. Hafeez N. West K. A. (2014). HSP90 inhibition enhances antimitotic drug-induced mitotic arrest and cell death in preclinical models of non-small cell lung cancer. *PLoS One* 9:e115228. 10.1371/journal.pone.0115228 25542032 PMC4277299

[B39] Parmigiani R. B. Xu W. S. Venta-Perez G. Erdjument-Bromage H. Yaneva M. Tempst P. (2008). HDAC6 is a specific deacetylase of peroxiredoxins and is involved in redox regulation. *Proc. Natl. Acad. Sci. U.S.A.* 105 9633–9638. 10.1073/pnas.0803749105 18606987 PMC2443817

[B40] Saji S. Kawakami M. Hayashi S. Yoshida N. Hirose M. Horiguchi S. (2005). Significance of HDAC6 regulation via estrogen signaling for cell motility and prognosis in estrogen receptor-positive breast cancer. *Oncogene* 24 4531–4539. 10.1038/sj.onc.1208646 15806142

[B41] Santo L. Hideshima T. Kung A. L. Tseng J. C. Tamang D. Yang M. (2012). Preclinical activity, pharmacodynamic, and pharmacokinetic properties of a selective HDAC6 inhibitor, ACY-1215, in combination with bortezomib in multiple myeloma. *Blood* 119 2579–2589. 10.1182/blood-2011-10-387365 22262760 PMC3337713

[B42] Sapra S. Bhalla Y. Sharma S. Singh G. Nepali K. (2013). Colchicine and its various physicochemical and biological aspects. *Med. Chem. Res.* 22 531–547. 10.1007/s00044-012-0077-z

[B43] Saunders L. R. Verdin E. (2007). Sirtuins: critical regulators at the crossroads between cancer and aging. *Oncogene* 26 5489–5504. 10.1038/sj.onc.1210616 17694089

[B44] Schrodinger L. (2010). *The PyMOL Molecular Graphics System, Version 1, 3r1.* Available at: http://www.pymol.org

[B45] Seidel C. Schnekenburger M. Dicato M. Diederich M. (2015). Histone deacetylase 6 in health and disease. *Epigenomics* 7 103–118. 10.2217/epi.14.69 25687470

[B46] Senapati J. Devasia A. J. Ganapule A. George L. Viswabandya A. (2014). Sorafenib induced hand foot skin rash in FLT3 ITD mutated acute myeloid leukemia-a case report and review of literature. *Mediterr. J. Hematol. Infect. Dis.* 6:e2014016. 10.4084/mjhid.2014.016 24678393 PMC3965723

[B47] Simoes-Pires C. A. Bertrand P. Cuendet M. (2017). Novel histone deacetylase 6 (HDAC6) selective inhibitors: a patent evaluation (WO2014181137). *Expert Opin. Ther. Pat.* 27 229–236. 10.1080/13543776.2017.1282945 28092474

[B48] Talevi A. (2015). Multi-target pharmacology: possibilities and limitations of the “skeleton key approach” from a medicinal chemist perspective. *Front. Pharmacol.* 6:205 10.3389/fphar.2015.00205 PMC4585027 26441661

[B49] Wang Y. Wallach J. Duane S. Wang Y. Wu J. Wang J. (2017). Developing selective histone deacetylases (HDACs) inhibitors through ebselen and analogs. *Drug Des. Dev. Ther.* 11 1369–1382. 10.2147/dddt.s124977 28496307 PMC5422321

[B50] Wang Y. Zhang H. Gigant B. Yu Y. Wu Y. Chen X. (2016). Structures of a diverse set of colchicine binding site inhibitors in complex with tubulin provide a rationale for drug discovery. *FEBS J.* 283 102–111. 10.1111/febs.13555 26462166

[B51] Widakowich C. de Castro G. Jr. de Azambuja E. Dinh P. Awada A. (2007). Review: side effects of approved molecular targeted therapies in solid cancers. *Oncologist* 12 1443–1455. 10.1634/theoncologist.12-12-1443 18165622

[B52] Yoon S. Eom G. H. (2016). HDAC and HDAC inhibitor: from cancer to cardiovascular diseases. *Chonnam Med. J.* 52 1–11. 10.4068/cmj.2016.52.1.1 26865995 PMC4742605

[B53] Zhang Y. Li N. Caron C. Matthias G. Hess D. Khochbin S. (2003). HDAC-6 interacts with and deacetylates tubulin and microtubules *in vivo*. *EMBO J.* 22 1168–1179. 10.1093/emboj/cdg115 12606581 PMC150348

